# Antenatal care and skilled delivery service utilisation in Somali pastoral communities of Eastern Ethiopia

**DOI:** 10.1111/tmi.13346

**Published:** 2019-12-09

**Authors:** A. Umer, J. Zinsstag, E. Schelling, R. Tschopp, J. Hattendof, K. Osman, M. Yuya, A. Ame, E. Zemp

**Affiliations:** ^1^ Jigjiga University Jigjiga Ethiopia; ^2^ Swiss Tropical and Public Health Institute Basel Switzerland; ^3^ University of Basel Basel Switzerland; ^4^ Armauer Hansen Research Institute Addis Ababa Ethiopia; ^5^ Dire Dawa University Dire Dawa Ethiopia

**Keywords:** maternal health, pastoral, antenatal care, skilled delivery service, santé maternelle, pastorale, soins prénatals, service qualifié d, accouchement

## Abstract

**Objective:**

To assess maternal health care service utilisation and associated factors in Somali pastoral communities of eastern Ethiopia.

**Methods:**

Community‐based cross‐sectional study complemented by qualitative assessments in Adadle district, Somali region, eastern Ethiopia, among 450 women in six *kebeles* from August to September 2016. Logistic regression was used to assess factors associated with antenatal care use and skilled delivery care use, controlling for confounders.

**Results:**

About 27% [95%CI 22.8–31.2%] of women used antenatal care, and 22.6% [95%CI 18.7–26.5%] received skilled delivery service. None of the respondents reported post‐natal care. About 43% reported that they had no knowledge of antenatal care, and 46% did not perceive delivery at a health facility as important. Pastoral lifestyle, husband’s educational status, women’s attitude towards health care service and financial support from the husband were significantly associated with antenatal care utilisation. Health professionals’ attitudes, perceptions of institutional delivery, antenatal care utilisation and information about exemptions from maternal health care fees were associated with skilled delivery service utilisation.

**Conclusion:**

Improving community awareness of antenatal care, employing female health professionals and culturally adapted guidelines could improve skilled delivery utilisation. In a patriarchal society, involving male partners in all maternal health issues is essential to increase use of maternal health services and to decrease maternal mortality.

## Abstract (fr)

**Objectif:**

Evaluer l'utilisation des services de soins de santé maternelle et les facteurs associés dans les communautés pastorales somaliennes dans l'est de l'Ethiopie.

**Méthodes:**

Etude transversale basée sur la communauté complétée par des évaluations qualitatives dans le district d'Adadle, région somalienne, dans l'est de l'Ethiopie, portant sur 450 femmes dans six *kebeles* d'août à septembre 2016. Une régression logistique a été utilisée pour évaluer les facteurs associés à l'utilisation des soins prénatals et à l'accouchement sous soins qualifiés, en ajustant pour les facteurs confusionnels.

**Résultats:**

27% [IC95%: 22.8–31.2%] des femmes ont utilisé des soins prénatals et 22,6% [IC95%: 18.7‐26.5%] ont reçu des services d'accouchement qualifiés. Aucune des répondantes n'a signalé des soins postnatals. 43% ont déclaré qu'elles n'avaient aucune connaissance des soins prénatals et 46% ne percevaient pas l'accouchement dans un établissement de santé comme important. Le mode de vie pastoral, le niveau d'éducation du mari, l'attitude des femmes à l'égard des services de santé et le soutien financier du mari étaient significativement associés à l'utilisation des soins prénatals. Les attitudes des professionnels de la santé, les perceptions de l'accouchement en établissement, l'utilisation des soins prénatals et les informations sur les exemptions des frais de soins de santé maternelle ont été associées à l'utilisation des services d'accouchement qualifiés.

**Conclusion:**

Améliorer la sensibilisation de la communauté aux soins prénatals, employer des professionnels de la santé féminins et des directives culturellement adaptées pourrait améliorer l'utilisation de l’accouchement qualifié. Dans une société patriarcale, la participation des partenaires masculins à toutes les questions de santé maternelle est essentielle pour accroître l'utilisation des services de santé maternelle et réduire la mortalité maternelle.

## Introduction

Maternal health services are a core component of universal health coverage (UHC) according to WHO 1, being part of the human right to equitable access to quality health care. Though this is a guiding principle to achieve Sustainable Development Goal (SDG target 3.1) 2, it does not seem to be adapted to pastoral communities across the globe. In the last two decades, global maternal mortality has decreased by 38%. Despite this progress, an estimated 295 000 maternal deaths occurred in 2017 3. The latest Ethiopian Demographic and Health Survey (EDHS) estimates 412 maternal deaths per 10 000 live births 4, which is far from the goal set by the Ethiopian reproductive health programme of 199 maternal deaths per 100 000 live births by 2020 5. Two‐thirds of maternal mortality in low and middle income countries occur due to direct obstetric complications 6. Antenatal care, skilled birth attendance at delivery and post‐natal care are critical ingredients in preventing maternal deaths 7, 8, 9. Despite this knowledge, in 2016 at national level, only 62% of women attended at least one antenatal care session, only 26 % of deliveries were assisted by a skilled birth attendant, and only 49% of women had their last birth protected against neonatal tetanus in Ethiopia 4. Maternal health service utilisation varies across different parts of the nine regions of Ethiopia. The percentage of women receiving skilled delivery service is 18% in the Somali region, where the majority of the population are pastoralist, but reaches 57% in the Tigray region, where almost all the population is agrarian 4. This variance in utilisation of antenatal and skilled delivery services has been linked to maternal socio‐economic status 10, couples’ literacy 11, maternal age, health professionals’ attitudes 8, 12 and distance to health facility 13. Barriers to the uptake of maternal health service are a combination of personal behaviours, health systems and geo‐demography 11, 14, 15, 16, 17.

In a pastoral setting, this combination is stronger due to the way of life and mobility 18, 19. Pastoral communities have very little access to health care 20, and their health is poor with regard to a number of conditions, ranging from vaccine‐preventable diseases to sexually transmitted diseases 21. Pastoral communities are marginalised in terms of health 22, infrastructure and social services 23. Maternal health is one of their top priority health problems 7, 24, 25, 26. To tackle this complex problem, the Ethiopian government introduced health extension workers 7, and later a volunteer women development army 27 was formed to deliver this service at community level. However, due to their way of life and mobility 16, their high illiteracy rates 28, and drawbacks of the policy itself, pastoral communities have very limited access to antenatal and skilled delivery services 24, 29, 30, 31.

Few studies depict comprehensive maternal health care through the course of antenatal care to delivery in pastoral communities 32, 33. It is paramount important to understand factors affecting maternal health care utilisation in pastoral communities, using quantitative and qualitative methods as the first step towards locally adapted policies. This study is part of the Jigjiga One Health Initiative (JOHI) project, a partnership between Jigjiga University (JJU) in Jigjiga, the Armauer Hansen Research Institute (AHRI) in Addis Ababa and the Swiss Tropical and Public Health Institute (Swiss TPH), in Basel.

## Methods

### Study area and period

Adadle district is located in the Somali region of Ethiopia. It has an estimated population of 100 000, of which 57 555 are males and 42 445 females. The district has 3 health centres serving 25 000, and six health posts serving 5000 people each. The inhabitants of the district, 75% pastorals with 99.9% being Muslim, belong to the Somali ethnic group. We conducted the study from July to September 2016.

### Study design and population

This community‐based, cross‐sectional study included women who had at least one child up to the age of three years or who were pregnant at the time of the study and their male partners. Women who were deaf mute were excluded. We checked recall bias by relating proportions of service utilisation to the time since the last delivery.

### Sample size and sampling technique

For the sample size calculation, we considered clustering of households within the study *kebele* (the smallest Ethiopian administration unit) assuming an intra‐class correlation coefficient of 0.2 and a prevalence of maternal health service utilisation of 10%. This resulted in 18 eligible women per cluster, yielding a total of 450 women with a standard error of 3 % 34.

Of the 15 *kebeles* in Adadle Woreda district, 6 were selected proportional to their size using a census list. We selected households by spinning a pencil. The data collectors then walked to the edge of the sub‐*kebele* into the direction to which the pencil pointed, numbering all households on the way. We chose a random number to identify one household as starting household for the cluster. Data collectors then continued to the right side until the targeted number of samples per cluster was reached.

### Data collection tools and procedure

We collected quantitative (socio‐demographic and obstetric characteristics) and qualitative data (bottlenecks of health seeking behaviour) by face‐to‐face interviews using structured and semi‐structured, pre‐tested questionnaires, respectively. The questionnaire was prepared in English and translated to Somali. For its validation, it was then back translated to English by a language expert. Health professionals fluent in the local language were employed for data collection and supervised by the investigator (A.U.) 35, 36.

### Data analysis

Data were double entered in the data base (Access 2003^®^, Microsoft, Redmond WA), compared with Epi‐Info™ (version 3.5.1, CDC, Atlanta, GA) and analysed by intercooled Stata^®^ 14, College Station, TX). We conducted bivariate and multivariate analyses. We checked correlations of predictive variables by calculating the Pearson’s correlation coefficients (*r*). At bivariate level, chi‐square testing was used to assess the association between utilisation of antenatal care, skilled birth attendants and further independent variables. Multivariate logistic regression models were fitted for antenatal and for skilled delivery service use, with scientifically plausible explanatory variables. Statistical significance was set at *P*‐values of less than 0.05. The strength of the association was assessed using the odds ratio with 95% confidence intervals. Recorded focus groups discussions were first transcribed and translated into English, and then reread and coded. Analysis of qualitative data was guided by qualitative content analysis using the data analysis software MAXqda12^®^ (VERBI GmbH, Berlin, Germany).

### Ethical clearance

Ethical clearance was obtained prior to initiation of the study from the ethics committees of Northwestern and Central Switzerland (EKNZ UBE‐req. 2016‐00204) and the Jigjiga University Ethical Review Board in Ethiopia. All participants were informed about the objective of the study, and verbal and written consent were obtained before the interview.

## Results

### Socio‐demographic characteristics of the respondents

Of 450 women who were asked to be interviewed, 97% participated in the study. Their mean age was 29.7 years (SD 7); 50.2% were aged between 25 and 34 years. Their husbands were considerably older with a mean age of 38.5 years (standard deviation 11.2 years), and 57% were aged 35 to 65 years (Table 1). A quarter of respondents were in polygamous marital unions.

**Table 1:** Socio‐demographic characteristics of respondents in Adadle district, Somali region, eastern Ethiopia, August, 2016

	Frequency (*n*)	Percentage (%)	95% confidence interval (%)
Lifestyle			
Pastoral	238 (438)	54.4	49.6–59.0
Agro‐pastoral	200 (438)	45.6	40.9–50.3
Age of mother			
15–24	96 (438)	21.9	18.0–25.7
25–34	220 (438)	50.2	45.5–54.9
35–49	122 (438)	27.8	23.6–32.0
Age of male partner			
15–24	23 (427)	5.3	3.2–7.5
25–34	155 (427)	35.5	31.7–40.8
35–49	176 (427)	40.3	36.5–45.8
50–65	60 (427)	15.8	10.7–17.3
>65	13 (427)	3.0	1.4–4.6
Union			
Monogamous	330	75.3	71.3–79.3
Polygamous	108	24.5	20.6–28.6
Job of mother			
Housewife	338 (438)	77.2	73.2–81.1
Pastoralist	85 (438)	19.4	15.7–23.1
Other	15 (438)	3.4	1.7–5.1
Job of male partner			
Pastoralist	238 (427)	54.9	51.0–60.4
Agro‐pastoralist	109 (427)	25.1	21.3–29.6
Merchant	39 (427)	9.1	6.4–11.8
Others	41 (427)	9.6	6.8–12.3
Educational status of women			
Cannot read and write	414 (438)	94.5	92.3–96.6
Educated	24 (438)	5.5	3.3–7.6
Educational status of husband			
Cannot read and write	364 (427)	83.3	81.8–88.6
1–8 grades	38 (427)	8.7	6.1–11.6
9–12 grades	35 (427)	8.0	5.5–10.7
Diploma	5 (427)	1.1	0.01–2.1
Family size			
1–4	94	21.5	17.6–25.3
5–9	268	61.1	56.6–65.7
>=10	76	17.4	13.8–20.8

The proportion reporting not to be able to read and write was 94.5% of women and 83.3% of men. 77.7% of the women said they were housewives and around 20.0% reported to be pastoralist (keep herds or livestock), whereas 54% of their male partners reported to be pastoralist and 25% said they were agro‐pastoral (with both pastoral and agrarian lifestyles).

### Obstetric characteristics and maternal health care utilisation

**Figure 1:**
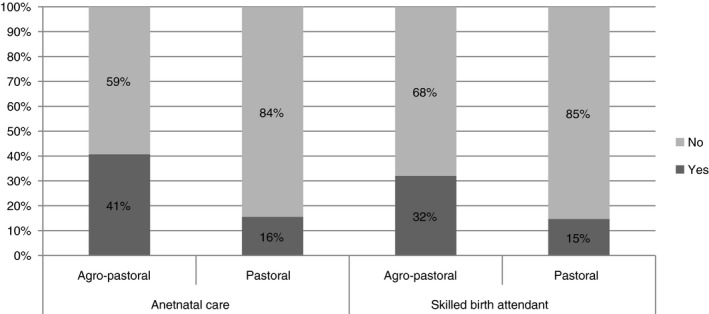
Antenatal and skilled delivery service utilisation among pastoral and agro‐pastoral in Adadle district, Somali region, eastern Ethiopia, August, 2016.

About 52.5% of the women were multipara with at least five pregnancies in their life, and 11.2 % were primipara. About 27% of the 438 women who participated (95% confidence interval (CI) 22.8–31.1%) used antenatal care, and 22.6% (95% CI 18.6–26.5%) had their delivery at a health facility with skilled birth attendants. Women from agro‐pastoral *kebeles* were more likely to use antenatal and skilled delivery services (Figure 1).

Of 119 women who had antenatal care for their last pregnancy, only 66 (55.4%; 95%CI (46.5–64.3%) gave birth at a health facility. Of all deliveries which took place at home, 41.7% (95%CI 36.4–46.9%) were assisted by a traditional birth attendant and 42.7% (95%CI 37.4–47.9%) by neighbours.

Among the 119 women who had antenatal care, 17.9% (95%CI 11.0–24.7%) had visited at least once; only 7.9% (95%CI 3.0–12.7%) had completed four visits as recommended by WHO. Asked about the reasons why they did not attend antenatal care, a third of the women said they did not know about it, 40.1% said that the health facility would be far away, and 17% said they were not sick.

Regarding the decision on the preferred place of delivery, in 47% of cases, the husband decided alone; in 42%, the decision was made jointly by husband and wife.

**Figure 2:**
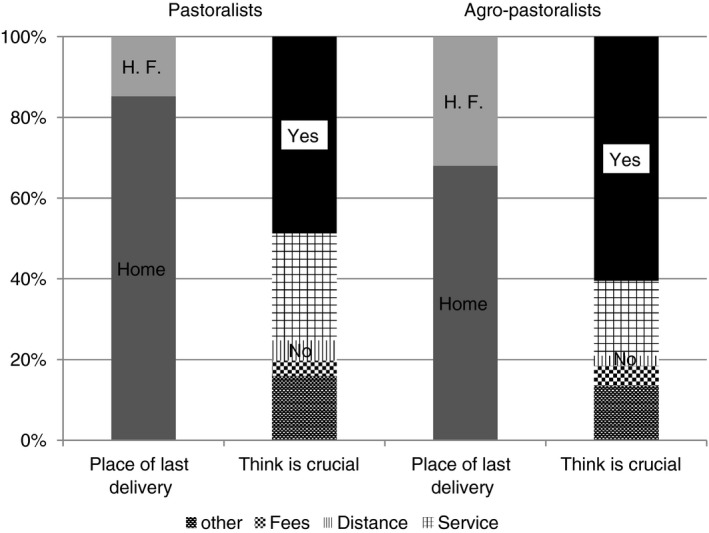
Place of last delivery and reasons for not perceiving institutional delivery as crucial in Adadle district, Somali region, eastern Ethiopia, August, 2016.

About 64% of women did not perceive skilled delivery service as crucial. About 27% of participants felt their privacy would not be kept. Additional reasons were the inconvenience of being referred to another health facility for delivery (15%), lack of individuals to take care of other children during their absence and presence of male attendants at the health facility. About 17% assumed they would not face delivery‐related complications. Home delivery is more common among pastoralists since only few women perceive skilled delivery service as crucial compared with agro‐pastoralists (Figure 2).

### Health service and health seeking behaviour of women

More than half (56.6%) of women reported that they had to travel more than one hour to the nearest health service to receive antenatal and skilled delivery services (Table 2). Every second woman reported that health professionals were not respectful, and 61.5% had no information about the fee exemption for community‐based maternal health care services.

**Table 2:** Health service use and health seeking behaviour of women in Adadle district, Somali region, eastern Ethiopia, August 2016

Characteristics	Frequency (*n*)	Percentage (%)	95% CI
Distance to the nearest health centre			
<1 h	190 (438)	43.4	38.8–48.0
≥1 h	248 (438)	56.6	52.0–61.2
Health professionals` attitude			
Welcoming	220 (438)	50.2	45.5–54.9
Not welcoming	218 (438)	49.8	45.1–54.5
Had information on maternal health care service fee exemption			
Yes	169 (438)	38.5	33.9–43.1
No	26 8(438)	61.5	56.9–66.1
Describe current health			
Very good	47 (438)	10.7	7.8–13.6
Good	279 (438)	63.7	59.2–68.2
Bad	109 (438)	24.9	20.9–28.9
Health professional visit			
Always	69 (438)	15.8	12.4–19.2
Sometimes	77 (438)	17.6	14.0–21.2
Often	62 (438)	14.2	10.9–17.5
Never	230 (438)	52.5	47.8–57.2
Reason of not visiting			
I am not sick	42 (230)	18.3	14.7–21.9
Health centre far	147 (230)	63.9	59.4–68.4
There is no service	41 (230)	17.4	13.8–21.0
Ask for help when get sick			
My husband	278 (438)	63.5	59.0–68.0
My mother	140 (438)	32.0	27.6–36.4
My mother‐in‐law	20 (438)	4.6	2.6–6.6
Preferred place to go when sick			
Traditional healer	32 (438)	7.3	4.9–9.7
Religious healer	196 (438)	44.8	40.1–49.5
Health facility	365 (438)	83.3	79.8–86.8
Other	40 (438)	4.6	2.6–6.6

Of all participants interviewed on their current health status, only 10.7% reported their current health status as very good, whereas 63.7% rated it as good and 25.6% as bad. About 52.6% said that they had not visited any health professional in the last three years. About 73% of women reported that they had have their husband’s permission to seek professional health support. About 63.5% chose their husband for help in the first instance; one‐third first asked their mother for help when they faced health problems. When feeling sick, 83% preferred to go to a health facility, whereas 17% preferred a religious healer.

In the focus group discussions (FGDs), almost all participants identified distance from health services, being healthy, lack of awareness about antenatal care, health professionals’ attitude, maternal health service fee, support from traditional birth attendants and neighbours as the major reasons for not utilising antenatal care. ‘*These days we share information, and all of us know the challenge that pregnant mother faces during childbirth from experience, for me for example my delivery is too fast whenever it comes. Even I cannot wait for an ambulance in most cases, so I don’t think it is important to go to health facility. If something is wrong with the normal process or it takes longer than usual to give birth then we demand the ambulance to take us to nearest health facility’*. (Woman, 31 years old).

FGD participants stated that independent decision making by the expectant mother is almost nil; family members such as the husband, mother‐in‐law, mother and relatives are involved in the decision on where to go for delivery. Female FGD participants argued among themselves about whether they get support from their partner during pregnancy and childbirth. Some women said their husband gave emotional support and looked after the children and did not only provide financial support. In the men’s FGD, almost all men agreed that they should provide support and encourage women to uptake or use the available maternal health care services. ‘*Men are better than they were in the past when it comes to maternal health. Even though this depends, in most cases, on men and his behaviour. Some men are good at caring for their pregnant wives, others might not even remember of her situation. Yet the majorities of men in our community are aware of and follow up the situation of their pregnant wives’*. TBA, 40 years.

### Factors associated with antenatal care use

In multivariate analyses (Table 3), the following factors were significantly associated with antenatal care use: lifestyle, educational status of male partner, financial support of partner during pregnancy, thinking that health facility delivery is crucial and knowing that maternal health service is free were associated with antenatal care utilisation. In pastoral communities, the use of antenatal care was more than three times less likely than that of women in agro‐pastoral communities [adjusted Odds ratio (aOR) 0.3 (0.2–0.6)]. Antenatal use among women whose partners have at least primary education was 3.5 times more likely than among those with an illiterate male partner [aOR 3.5 (1.4–7.8)]. Also, attendance of antenatal care was more likely among women who think that health institution delivery is crucial than among women who did not think so [aOR 2.8 (1.3–5.9)], and among women who had information on maternal health care service fee exemption than among those who did not have this information [aOR 2.6 (1.3–4.9)]. Women who had financial support from their male partner were also more likely to use antenatal care than those who did not [aOR 23.3 (11.0–49.5)].

**Table 3:** Factors associated with antenatal care service use of women, Adadle district, Somali region, eastern Ethiopia, August, 2016

Variables		Antenatal care use			
		Frequency (n)	Column (%)	Crude OR 95 % CI	Adjusted OR 95 % CI
Lifestyle	Agro‐pastoral	200	45.7	1	1
	Pastoral	238	54.3	0.3 (0.2–0.4)	0.3 (0.2–0.6)***
Age of mother	15–24	96	21.9	1	1
	25–34	220	50.2	1.2 (0.7–2.0)	1.8 (0.5–2.7)
	35–50	122	27.8	0.9 (0.5–1.6)	1.2 (0.5–3.0)
Educational status of male partner	Cannot read and write	364	83.3	1	1
	Can read and write	73	16.7	11.1 (6.3–19.8)***	3.5 (1.4–7.8)**
Financially supported during pregnancy	No	340	77.6	1	1
	Yes	98	22.4	43.1 (22.8–81.6)***	23.3 (11.0–49.5)***
Thinks health facility delivery is crucial	No	201	45.8	1	1
	Yes	237	54.2	7.9 (4.5–14.0)***	2.8 (1.3–5.9)**
Distance to health facility	>=1 h	190	43.4	1	1
	<1 h	248	56.6	7.8 (3.6–16.5)***	2.3 ( 0.9–6.0)
Had information about maternal health service fee exemption	No	268	61.5	1	1
	Yes	169	38.5	3.4 (2.2–5.3)***	2.6 (1.3–4.9)*

* *P*‐value ≤ 0.05.

** *P*‐value < 0.01.

*** *P*‐value < 0.001.

### Factors associated with skilled delivery service utilisation

Previous antenatal care use, belief that health facility delivery is crucial, reported attitude of health professionals and information on maternal health service fee exemption were positively associated with skilled birth attendant utilisation, whereas women who were illiterate were less likely to be assisted by skilled birth attendants (Table 4).

**Table 4:** Factors associated with skilled delivery service use of women, Adadle district, Somali region, Ethiopia, August, 2016

Characteristics		Skilled delivery service use			
		Frequency	Column (%)	Crude OR (95% CI)	Adjusted OR (95%CI)
Lifestyle	Pastoral	238	45.7	1	1
	Agro‐pastoral	200	54.3	0.4 (0.2–0.9)	0.9 (0.3–1.1)
Age of mother	15–24	96	21.9	1	1
	25–34	220	50.2	0.9 (0.5–1.7)	0.9 (0.4–1.8)
	35–50	122	27.8	1.0 (0.5–1.9)	1.2 (0.6–2.8)
Male partner education level	Illiterate	364	83.3	1	1
	Can read and write	73	16.7	5.0 (2.9–8.5)	1.4 (0.7–2.7)
Attended antenatal during last pregnancy	No	319	73	1	1
	Yes	118	27	11 (6.6–18.4)	3.2 (1.7–6.2)***
Do you think delivery at health facility is crucial	No	201	45.8	1	1
	Yes	237	54.2	8.2 (4.4–5.3)	3.0 (1.6–6.0)***
Distance to health facility	>=1 h	190	43.4	1	1
	<1 h	248	56.6	7.5 (4.6–2.2)	0.7 (0.3–1.8)
Health professional attitude	Not welcoming	257	58.6	1	1
	Welcoming	181	41.3	3.0 (2.3–3.9)	1.9 (1.4–6.7)**
Information about health service fee exemption	No	269	61.5	1	1
	Yes	169	38.5	3.6 (2.3–5.8)	1.8 (1.3–4.4)*

* *P*‐value ≤ 0.05.

** *P*‐value < 0.01.

*** *P*‐value < 0.001.

Women who had attended antenatal care at least one time were more than 3 times more likely to be assisted by skilled birth attendants than women who did not attended antenatal care [aOR 3.2; 95% CI 1.7–6.2]. Similarly, to the use of skilled delivery was threefold among women who think that health facility delivery is crucial aOR 3.0 (1.6–6.0)] compared with women who think that this is not crucial. Women who had information on maternal health care service fee exemption were 1.8 times more likely to choose delivery by skilled birth attendants than women who did not have this information [aOR 1.8 (1.3–4.4)]. Women who felt that health professionals’ attitudes towards her was welcoming were twice as likely to utilise skilled delivery care service as women who thought or had heard that the attitudes of health workers were not respectful [aOR 1.9 (1.4–6.7)].

## Discussion

Antenatal care and skilled delivery service use are low, at rates of 27% and 22.6%, respectively. Nobody used post‐natal delivery service. According to EDHS (2016), 67% of women attended at least one antenatal care while only 26% received skilled delivery service which is higher than the utilisation rate in present study 4. Both pastoral (83.5%) and agro‐pastoral (86%) communities in the study district had a high rate of home delivery. Health professionals should be better trained in counselling skills during antenatal care. Maternal health service utilisation is known to be associated with male partner support, economic dependency, literacy and mobility of women, as shown in a study conducted in the Afar region, Ethiopia 19 and EDHS. A similar finding is reported in linking antenatal care use with the socio‐economic of the women in different parts of low‐income countries 37. A recent study from Kenya shows that as the educational level of a couple increases, so does the use of antenatal care 24.

To further investigate the barriers of antenatal care and skilled delivery use and enable designing context‐specific interventions to overcome these challenges, a qualitative method was used. Barriers to use of antenatal care are a combination of personal behaviour, health systems and lifestyles. Skilled delivery care use was significantly associated with women’s perception of professionals’ attitude. Focus group participants mentioned concerns that most health professionals are not welcoming. The study from Afar region also shows that around 85% of pastoral women do not feel comfortable with male health professionals 32.

With regard to bottlenecks of antenatal and skilled delivery, many respondents (46%) reported that the health facility delivery is not crucial as the main reason for not using antenatal care. Other reasons for low utilisation of antenatal care are lack of awareness and autonomy decision making in health seeking. In pastoral community, as a norm, women ask their male partner to seek health care. In our study, half of the respondents have to ask for permission for health care. Thus, in patriarchal communities, involvement of male partner is crucial, confirming the findings on maternal health care utilisation in a nomadic Sudanese community 18. Women who were supported by their male partner had 80% more chance of antenatal care use than those without male support. Lack of awareness about antenatal care, misinterpretation or linking it with tetanus toxoid are factors affecting antenatal care use. A simply stated: ‘I am not sick why should I go to health facility?’. Lack of appropriate awareness on antenatal care is cited as the main factor preventing women from use of antenatal care in other studies also 19, 32. This implies inadequate quality of services provided by health extension workers in pastoral communities 38.

The most commonly reported (46%) reason for home delivery is that it is not seen as crucial. There is a need for awareness creation in the community at large, to achieve the Ethiopia reproductive health programme goal by 2020. Our study is in line with the findings from other pastoral communities showing that pastoral women prefer traditional birth attendants and healers and only travel to health facilities for complicated cases 39, 40, 41. Lack of privacy is mentioned as one of the most underreported but most important reasons for not using skilled delivery service (27% (95%CI 22.8–31.1%). This result is in line with other work conducted on maternal health both in pastoral and settled agrarian communities 18, 42, 43, 44. To improve maternal health care, local female health professionals should be included in designing population‐specific interventions. Individual behaviour is crucial in antenatal and skilled delivery service use 45, 46, 47, 48.

A strength of this study is that it used both quantitative and qualitative methods. It did not, however, cover the health professionals’ perspective or the quality of health services, which should also be included in further research on maternal health service utilisation.

## Conclusion

In view of the low maternal health care utilisation in the study area, nationwide health care plans are needed that take pastoral communities into account and make health care more accessible to them. Improving community awareness of antenatal care, employing female health professionals and culturally adapted guidelines could improve skilled delivery utilisation. At all levels, awareness of the importance of maternal health care must be raised. In a patriarchal society, involving male partners in all maternal health issues is essential to increase use of maternal health services and to decrease maternal mortality.
